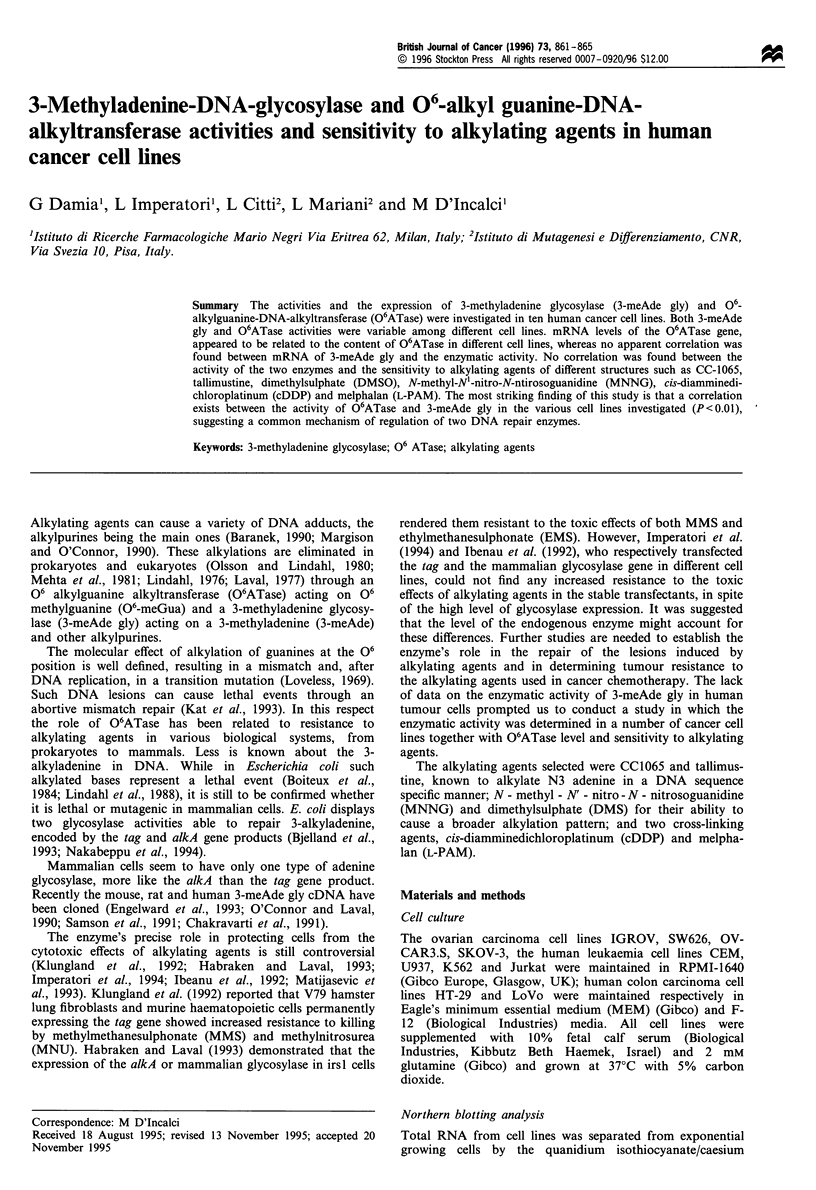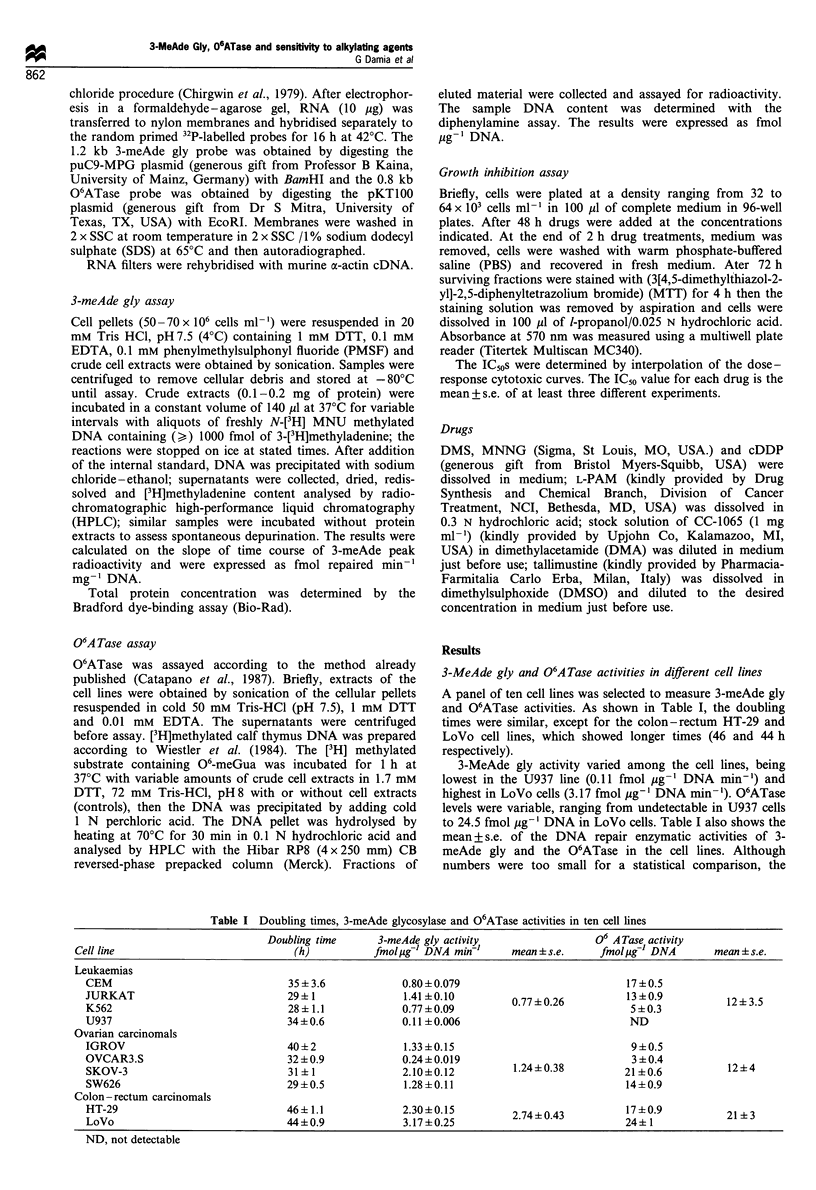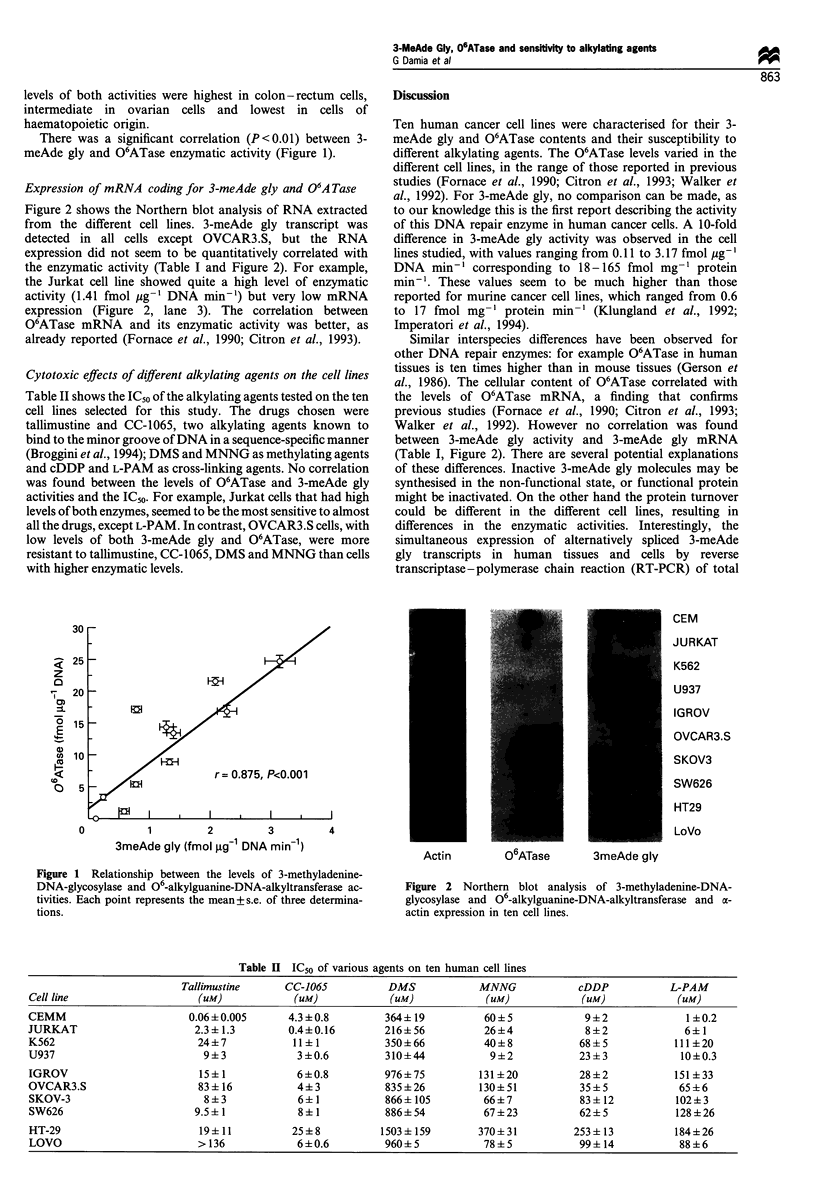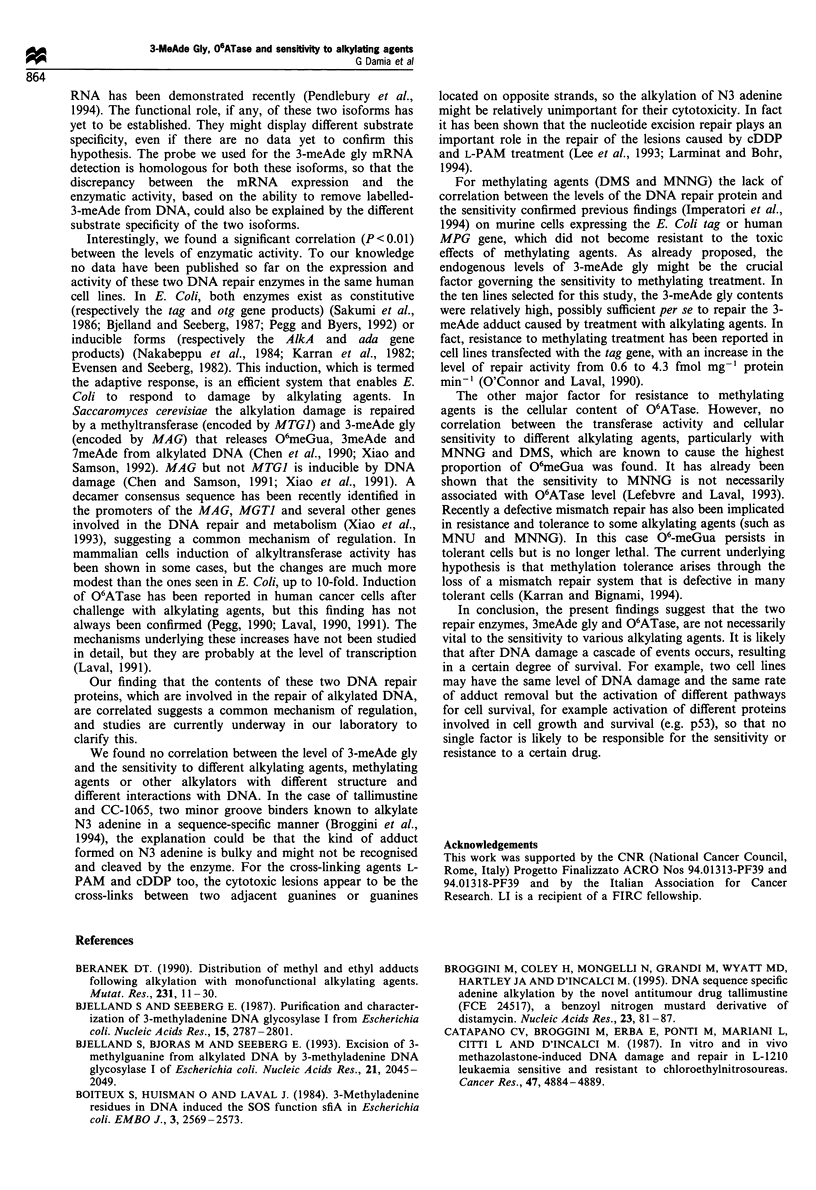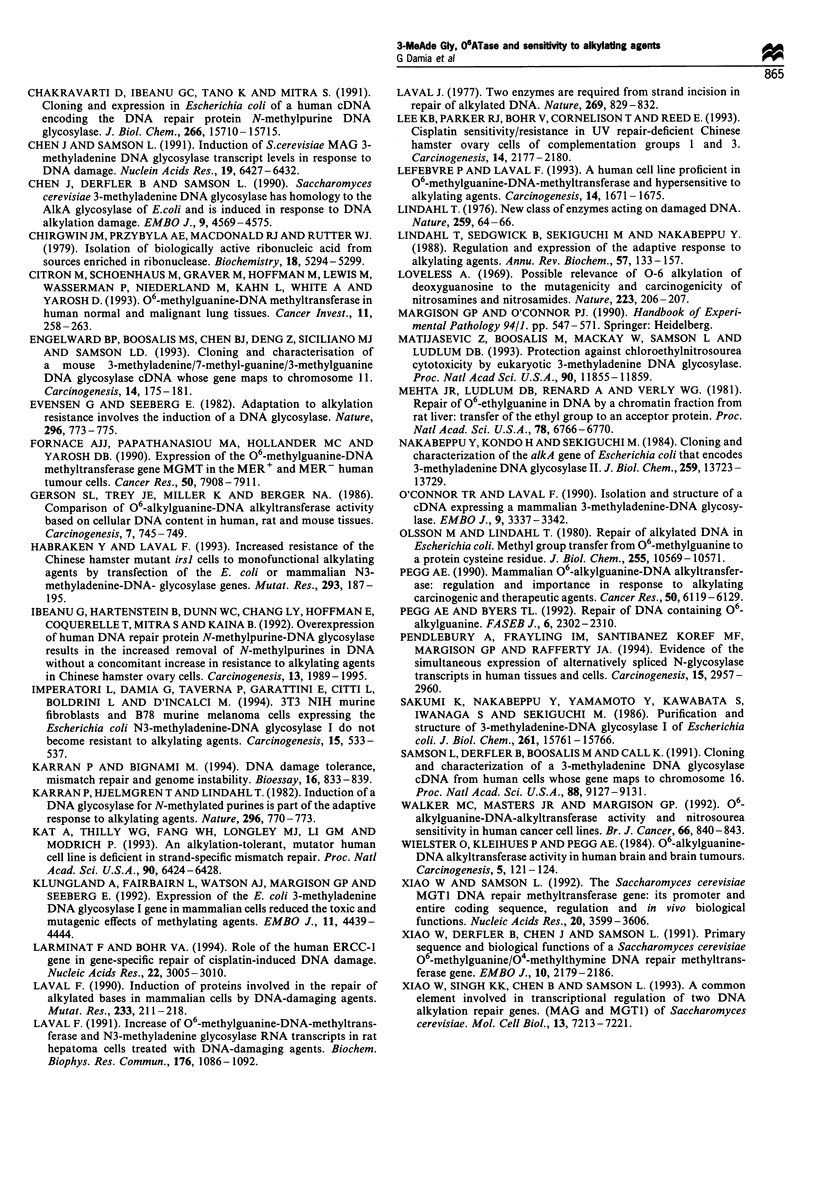# 3-methyladenine-DNA-glycosylase and O6-alkyl guanine-DNA-alkyltransferase activities and sensitivity to alkylating agents in human cancer cell lines.

**DOI:** 10.1038/bjc.1996.153

**Published:** 1996-04

**Authors:** G. Damia, L. Imperatori, L. Citti, L. Mariani, M. D'Incalci

**Affiliations:** Istituto di Ricerche Farmacologiche Mario Negri, Milan, Italy.

## Abstract

**Images:**


British Journal of Cancer (1996) 73, 861-865
© 1996 Stockton Press All rights reserved 0007-0920/96 $12.00

### 3-Methyladenine-DNA-glycosylase and O$^6$-alkyl guanine-DNA-alkyltransferase activities and sensitivity to alkylating agents in human cancer cell lines

G Damia[1], L Imperatori[1], L Citti[2], L Mariani[2] and M D'Incalci[1]

[1]*Istituto di Ricerche Farmacologiche Mario Negri Via Eritrea 62, Milan, Italy;* [2]*Istituto di Mutagenesi e Differenziamento, CNR, Via Svezia 10, Pisa, Italy.*

**Summary** The activities and the expression of 3-methyladenine glycosylase (3-meAde gly) and O$^6$-alkylguanine-DNA-alkyltransferase (O$^6$ATase) were investigated in ten human cancer cell lines. Both 3-meAde gly and O$^6$ATase activities were variable among different cell lines. mRNA levels of the O$^6$ATase gene, appeared to be related to the content of O$^6$ATase in different cell lines, whereas no apparent correlation was found between mRNA of 3-meAde gly and the enzymatic activity. No correlation was found between the activity of the two enzymes and the sensitivity to alkylating agents of different structures such as CC-1065, tallimustine, dimethylsulphate (DMSO), *N*-methyl-*N*$^1$-nitro-*N*-ntirosoguanidine (MNNG), *cis*-diamminedichloroplatinum (cDDP) and melphalan (L-PAM). The most striking finding of this study is that a correlation exists between the activity of O$^6$ATase and 3-meAde gly in the various cell lines investigated ($P<0.01$), suggesting a common mechanism of regulation of two DNA repair enzymes.

**Keywords:** 3-methyladenine glycosylase; O$^6$ ATase; alkylating agents

Alkylating agents can cause a variety of DNA adducts, the alkylpurines being the main ones (Baranek, 1990; Margison and O'Connor, 1990). These alkylations are eliminated in prokaryotes and eukaryotes (Olsson and Lindahl, 1980; Mehta *et al.*, 1981; Lindahl, 1976; Laval, 1977) through an O$^6$ alkylguanine alkyltransferase (O$^6$ATase) acting on O$^6$ methylguanine (O$^6$-meGua) and a 3-methyladenine glycosylase (3-meAde gly) acting on a 3-methyladenine (3-meAde) and other alkylpurines.

The molecular effect of alkylation of guanines at the O$^6$ position is well defined, resulting in a mismatch and, after DNA replication, in a transition mutation (Loveless, 1969). Such DNA lesions can cause lethal events through an abortive mismatch repair (Kat *et al.*, 1993). In this respect the role of O$^6$ATase has been related to resistance to alkylating agents in various biological systems, from prokaryotes to mammals. Less is known about the 3-alkyladenine in DNA. While in *Escherichia coli* such alkylated bases represent a lethal event (Boiteux *et al.*, 1984; Lindahl *et al.*, 1988), it is still to be confirmed whether it is lethal or mutagenic in mammalian cells. *E. coli* displays two glycosylase activities able to repair 3-alkyladenine, encoded by the *tag* and *alkA* gene products (Bjelland *et al.*, 1993; Nakabeppu *et al.*, 1994).

Mammalian cells seem to have only one type of adenine glycosylase, more like the *alkA* than the *tag* gene product. Recently the mouse, rat and human 3-meAde gly cDNA have been cloned (Engelward *et al.*, 1993; O'Connor and Laval, 1990; Samson *et al.*, 1991; Chakravarti *et al.*, 1991).

The enzyme's precise role in protecting cells from the cytotoxic effects of alkylating agents is still controversial (Klungland *et al.*, 1992; Habraken and Laval, 1993; Imperatori *et al.*, 1994; Ibeanu *et al.*, 1992; Matijasevic *et al.*, 1993). Klungland *et al.* (1992) reported that V79 hamster lung fibroblasts and murine haematopoietic cells permanently expressing the *tag* gene showed increased resistance to killing by methylmethanesulphonate (MMS) and methylnitrosurea (MNU). Habraken and Laval (1993) demonstrated that the expression of the *alkA* or mammalian glycosylase in irs1 cells rendered them resistant to the toxic effects of both MMS and ethylmethanesulphonate (EMS). However, Imperatori *et al.* (1994) and Ibenau *et al.* (1992), who respectively transfected the *tag* and the mammalian glycosylase gene in different cell lines, could not find any increased resistance to the toxic effects of alkylating agents in the stable transfectants, in spite of the high level of glycosylase expression. It was suggested that the level of the endogenous enzyme might account for these differences. Further studies are needed to establish the enzyme's role in the repair of the lesions induced by alkylating agents and in determining tumour resistance to the alkylating agents used in cancer chemotherapy. The lack of data on the enzymatic activity of 3-meAde gly in human tumour cells prompted us to conduct a study in which the enzymatic activity was determined in a number of cancer cell lines together with O$^6$ATase level and sensitivity to alkylating agents.

The alkylating agents selected were CC1065 and tallimustine, known to alkylate N3 adenine in a DNA sequence specific manner; *N* - methyl - *N'* - nitro - *N* - nitrosoguanidine (MNNG) and dimethylsulphate (DMS) for their ability to cause a broader alkylation pattern; and two cross-linking agents, *cis*-diamminedichloroplatinum (cDDP) and melphalan (L-PAM).

### Materials and methods

### *Cell culture*

The ovarian carcinoma cell lines IGROV, SW626, OV-CAR3.S, SKOV-3, the human leukaemia cell lines CEM, U937, K562 and Jurkat were maintained in RPMI-1640 (Gibco Europe, Glasgow, UK); human colon carcinoma cell lines HT-29 and LoVo were maintained respectively in Eagle's minimum essential medium (MEM) (Gibco) and F-12 (Biological Industries) media. All cell lines were supplemented with 10% fetal calf serum (Biological Industries, Kibbutz Beth Haemek, Israel) and 2 mM glutamine (Gibco) and grown at 37°C with 5% carbon dioxide.

### *Northern blotting analysis*

Total RNA from cell lines was separated from exponential growing cells by the quanidium isothiocyanate/caesium

Correspondence: M D'Incalci
Received 18 August 1995; revised 13 November 1995; accepted 20 November 1995

chloride procedure (Chirgwin et al., 1979). After electrophoresis in a formaldehyde-agarose gel, RNA (10 μg) was transferred to nylon membranes and hybridised separately to the random primed $^{32}$P-labelled probes for 16 h at 42°C. The 1.2 kb 3-meAde gly probe was obtained by digesting the puC9-MPG plasmid (generous gift from Professor B Kaina, University of Mainz, Germany) with *Bam*HI and the 0.8 kb O$^6$ATase probe was obtained by digesting the pKT100 plasmid (generous gift from Dr S Mitra, University of Texas, TX, USA) with EcoRI. Membranes were washed in 2 × SSC at room temperature in 2 × SSC /1% sodium dodecyl sulphate (SDS) at 65°C and then autoradiographed.

RNA filters were rehybridised with murine α-actin cDNA.

### *3-meAde gly assay*

Cell pellets (50-70 × $10^6$ cells $ml^{-1}$) were resuspended in 20 mM Tris HCl, pH 7.5 (4°C) containing 1 mM DTT, 0.1 mM EDTA, 0.1 mM phenylmethylsulphonyl fluoride (PMSF) and crude cell extracts were obtained by sonication. Samples were centrifuged to remove cellular debris and stored at −80°C until assay. Crude extracts (0.1-0.2 mg of protein) were incubated in a constant volume of 140 μl at 37°C for variable intervals with aliquots of freshly *N*-[$^3$H] MNU methylated DNA containing (⩾) 1000 fmol of 3-[$^3$H]methyladenine; the reactions were stopped on ice at stated times. After addition of the internal standard, DNA was precipitated with sodium chloride-ethanol; supernatants were collected, dried, redissolved and [$^3$H]methyladenine content analysed by radiochromatographic high-performance liquid chromatography (HPLC); similar samples were incubated without protein extracts to assess spontaneous depurination. The results were calculated on the slope of time course of 3-meAde peak radioactivity and were expressed as fmol repaired $min^{-1}$ $mg^{-1}$ DNA.

Total protein concentration was determined by the Bradford dye-binding assay (Bio-Rad).

### *O$^6$ATase assay*

O$^6$ATase was assayed according to the method already published (Catapano et al., 1987). Briefly, extracts of the cell lines were obtained by sonication of the cellular pellets resuspended in cold 50 mM Tris-HCl (pH 7.5), 1 mM DTT and 0.01 mM EDTA. The supernatants were centrifuged before assay. [$^3$H]methylated calf thymus DNA was prepared according to Wiestler et al. (1984). The [$^3$H] methylated substrate containing O$^6$-meGua was incubated for 1 h at 37°C with variable amounts of crude cell extracts in 1.7 mM DTT, 72 mM Tris-HCl, pH 8 with or without cell extracts (controls), then the DNA was precipitated by adding cold 1 N perchloric acid. The DNA pellet was hydrolysed by heating at 70°C for 30 min in 0.1 N hydrochloric acid and analysed by HPLC with the Hibar RP8 (4 × 250 mm) CB reversed-phase prepacked column (Merck). Fractions of eluted material were collected and assayed for radioactivity. The sample DNA content was determined with the diphenylamine assay. The results were expressed as fmol $μg^{-1}$ DNA.

### *Growth inhibition assay*

Briefly, cells were plated at a density ranging from 32 to 64 × $10^3$ cells $ml^{-1}$ in 100 μl of complete medium in 96-well plates. After 48 h drugs were added at the concentrations indicated. At the end of 2 h drug treatments, medium was removed, cells were washed with warm phosphate-buffered saline (PBS) and recovered in fresh medium. Ater 72 h surviving fractions were stained with (3[4,5-dimethylthiazol-2-yl]-2,5-diphenyltetrazolium bromide) (MTT) for 4 h then the staining solution was removed by aspiration and cells were dissolved in 100 μl of *l*-propanol/0.025 N hydrochloric acid. Absorbance at 570 nm was measured using a multiwell plate reader (Titertek Multiscan MC340).

The $IC_{50}$s were determined by interpolation of the dose-response cytotoxic curves. The $IC_{50}$ value for each drug is the mean ± s.e. of at least three different experiments.

### *Drugs*

DMS, MNNG (Sigma, St Louis, MO, USA.) and cDDP (generous gift from Bristol Myers-Squibb, USA) were dissolved in medium; L-PAM (kindly provided by Drug Synthesis and Chemical Branch, Division of Cancer Treatment, NCI, Bethesda, MD, USA) was dissolved in 0.3 N hydrochloric acid; stock solution of CC-1065 (1 mg $ml^{-1}$) (kindly provided by Upjohn Co, Kalamazoo, MI, USA) in dimethylacetamide (DMA) was diluted in medium just before use; tallimustine (kindly provided by Pharmacia-Farmitalia Carlo Erba, Milan, Italy) was dissolved in dimethylsulphoxide (DMSO) and diluted to the desired concentration in medium just before use.

### Results

### *3-MeAde gly and O$^6$ATase activities in different cell lines*

A panel of ten cell lines was selected to measure 3-meAde gly and O$^6$ATase activities. As shown in Table I, the doubling times were similar, except for the colon-rectum HT-29 and LoVo cell lines, which showed longer times (46 and 44 h respectively).

3-MeAde gly activity varied among the cell lines, being lowest in the U937 line (0.11 fmol $μg^{-1}$ DNA $min^{-1}$) and highest in LoVo cells (3.17 fmol $μg^{-1}$ DNA $min^{-1}$). O$^6$ATase levels were variable, ranging from undetectable in U937 cells to 24.5 fmol $μg^{-1}$ DNA in LoVo cells. Table I also shows the mean ± s.e. of the DNA repair enzymatic activities of 3-meAde gly and the O$^6$ATase in the cell lines. Although numbers were too small for a statistical comparison, the

**Table I** Doubling times, 3-meAde glycosylase and O$^6$ATase activities in ten cell lines

| *Cell line* | *Doubling time (h)* | *3-meAde gly activity fmol $μg^{-1}$ DNA $min^{-1}$* | *mean ± s.e.* | *O$^6$ ATase activity fmol $μg^{-1}$ DNA* | *mean ± s.e.* |
|---|---|---|---|---|---|
| Leukaemias | | | | | |
| CEM | 35 ± 3.6 | 0.80 ± 0.079 | 0.77 ± 0.26 | 17 ± 0.5 | 12 ± 3.5 |
| JURKAT | 29 ± 1 | 1.41 ± 0.10 | | 13 ± 0.9 | |
| K562 | 28 ± 1.1 | 0.77 ± 0.09 | | 5 ± 0.3 | |
| U937 | 34 ± 0.6 | 0.11 ± 0.006 | | ND | |
| Ovarian carcinomals | | | | | |
| IGROV | 40 ± 2 | 1.33 ± 0.15 | 1.24 ± 0.38 | 9 ± 0.5 | 12 ± 4 |
| OVCAR3.S | 32 ± 0.9 | 0.24 ± 0.019 | | 3 ± 0.4 | |
| SKOV-3 | 31 ± 1 | 2.10 ± 0.12 | | 21 ± 0.6 | |
| SW626 | 29 ± 0.5 | 1.28 ± 0.11 | | 14 ± 0.9 | |
| Colon-rectum carcinomals | | | | | |
| HT-29 | 46 ± 1.1 | 2.30 ± 0.15 | 2.74 ± 0.43 | 17 ± 0.9 | 21 ± 3 |
| LoVo | 44 ± 0.9 | 3.17 ± 0.25 | | 24 ± 1 | |

ND, not detectable levels of both activities were highest in colon–rectum cells, intermediate in ovarian cells and lowest in cells of haematopoietic origin.

There was a significant correlation ($P < 0.01$) between 3-meAde gly and $O^6$ATase enzymatic activity (Figure 1).

*Expression of mRNA coding for 3-meAde gly and $O^6$ATase*

Figure 2 shows the Northern blot analysis of RNA extracted from the different cell lines. 3-meAde gly transcript was detected in all cells except OVCAR3.S, but the RNA expression did not seem to be quantitatively correlated with the enzymatic activity (Table I and Figure 2). For example, the Jurkat cell line showed quite a high level of enzymatic activity (1.41 fmol $\mu g^{-1}$ DNA $min^{-1}$) but very low mRNA expression (Figure 2, lane 3). The correlation between $O^6$ATase mRNA and its enzymatic activity was better, as already reported (Fornace *et al.*, 1990; Citron *et al.*, 1993).

*Cytotoxic effects of different alkylating agents on the cell lines*

Table II shows the $IC_{50}$ of the alkylating agents tested on the ten cell lines selected for this study. The drugs chosen were tallimustine and CC-1065, two alkylating agents known to bind to the minor groove of DNA in a sequence-specific manner (Broggini *et al.*, 1994); DMS and MNNG as methylating agents and cDDP and L-PAM as cross-linking agents. No correlation was found between the levels of $O^6$ATase and 3-meAde gly activities and the $IC_{50}$. For example, Jurkat cells that had high levels of both enzymes, seemed to be the most sensitive to almost all the drugs, except L-PAM. In contrast, OVCAR3.S cells, with low levels of both 3-meAde gly and $O^6$ATase, were more resistant to tallimustine, CC-1065, DMS and MNNG than cells with higher enzymatic levels.

### Discussion

Ten human cancer cell lines were characterised for their 3-meAde gly and $O^6$ATase contents and their susceptibility to different alkylating agents. The $O^6$ATase levels varied in the different cell lines, in the range of those reported in previous studies (Fornace *et al.*, 1990; Citron *et al.*, 1993; Walker *et al.*, 1992). For 3-meAde gly, no comparison can be made, as to our knowledge this is the first report describing the activity of this DNA repair enzyme in human cancer cells. A 10-fold difference in 3-meAde gly activity was observed in the cell lines studied, with values ranging from 0.11 to 3.17 fmol $\mu g^{-1}$ DNA $min^{-1}$ corresponding to 18–165 fmol $mg^{-1}$ protein $min^{-1}$. These values seem to be much higher than those reported for murine cancer cell lines, which ranged from 0.6 to 17 fmol $mg^{-1}$ protein $min^{-1}$ (Klungland *et al.*, 1992; Imperatori *et al.*, 1994).

Similar interspecies differences have been observed for other DNA repair enzymes: for example $O^6$ATase in human tissues is ten times higher than in mouse tissues (Gerson *et al.*, 1986). The cellular content of $O^6$ATase correlated with the levels of $O^6$ATase mRNA, a finding that confirms previous studies (Fornace *et al.*, 1990; Citron *et al.*, 1993; Walker *et al.*, 1992). However no correlation was found between 3-meAde gly activity and 3-meAde gly mRNA (Table I, Figure 2). There are several potential explanations of these differences. Inactive 3-meAde gly molecules may be synthesised in the non-functional state, or functional protein might be inactivated. On the other hand the protein turnover could be different in the different cell lines, resulting in differences in the enzymatic activities. Interestingly, the simultaneous expression of alternatively spliced 3-meAde gly transcripts in human tissues and cells by reverse transcriptase–polymerase chain reaction (RT-PCR) of total

**Figure 1** Relationship between the levels of 3-methyladenine-DNA-glycosylase and $O^6$-alkylguanine-DNA-alkyltransferase activities. Each point represents the mean ± s.e. of three determinations.

**Figure 2** Northern blot analysis of 3-methyladenine-DNA-glycosylase and $O^6$-alkylguanine-DNA-alkyltransferase and α-actin expression in ten cell lines.

**Table II** $IC_{50}$ of various agents on ten human cell lines

| Cell line | Tallimustine (uM) | CC-1065 (uM) | DMS (uM) | MNNG (uM) | cDDP (uM) | L-PAM (uM) |
|---|---|---|---|---|---|---|
| CEMM | 0.06 ± 0.005 | 4.3 ± 0.8 | 364 ± 19 | 60 ± 5 | 9 ± 2 | 1 ± 0.2 |
| JURKAT | 2.3 ± 1.3 | 0.4 ± 0.16 | 216 ± 56 | 26 ± 4 | 8 ± 2 | 6 ± 1 |
| K562 | 24 ± 7 | 11 ± 1 | 350 ± 66 | 40 ± 8 | 68 ± 5 | 111 ± 20 |
| U937 | 9 ± 3 | 3 ± 0.6 | 310 ± 44 | 9 ± 2 | 23 ± 3 | 10 ± 0.3 |
| IGROV | 15 ± 1 | 6 ± 0.8 | 976 ± 75 | 131 ± 20 | 28 ± 2 | 151 ± 33 |
| OVCAR3.S | 83 ± 16 | 4 ± 3 | 835 ± 26 | 130 ± 51 | 35 ± 5 | 65 ± 6 |
| SKOV-3 | 8 ± 3 | 6 ± 1 | 866 ± 105 | 66 ± 7 | 83 ± 12 | 102 ± 3 |
| SW626 | 9.5 ± 1 | 8 ± 1 | 886 ± 54 | 67 ± 23 | 62 ± 5 | 128 ± 26 |
| HT-29 | 19 ± 11 | 25 ± 8 | 1503 ± 159 | 370 ± 31 | 253 ± 13 | 184 ± 26 |
| LOVO | >136 | 6 ± 0.6 | 960 ± 5 | 78 ± 5 | 99 ± 14 | 88 ± 6 |

RNA has been demonstrated recently (Pendlebury *et al.*, 1994). The functional role, if any, of these two isoforms has yet to be established. They might display different substrate specificity, even if there are no data yet to confirm this hypothesis. The probe we used for the 3-meAde gly mRNA detection is homologous for both these isoforms, so that the discrepancy between the mRNA expression and the enzymatic activity, based on the ability to remove labelled-3-meAde from DNA, could also be explained by the different substrate specificity of the two isoforms.

Interestingly, we found a significant correlation ($P < 0.01$) between the levels of enzymatic activity. To our knowledge no data have been published so far on the expression and activity of these two DNA repair enzymes in the same human cell lines. In *E. Coli*, both enzymes exist as constitutive (respectively the *tag* and *otg* gene products) (Sakumi *et al.*, 1986; Bjelland and Seeberg, 1987; Pegg and Byers, 1992) or inducible forms (respectively the *AlkA* and *ada* gene products) (Nakabeppu *et al.*, 1984; Karran *et al.*, 1982; Evensen and Seeberg, 1982). This induction, which is termed the adaptive response, is an efficient system that enables *E. Coli* to respond to damage by alkylating agents. In *Saccaromyces cerevisiae* the alkylation damage is repaired by a methyltransferase (encoded by *MTG1*) and 3-meAde gly (encoded by *MAG*) that releases $O^6$meGua, 3meAde and 7meAde from alkylated DNA (Chen *et al.*, 1990; Xiao and Samson, 1992). *MAG* but not *MTG1* is inducible by DNA damage (Chen and Samson, 1991; Xiao *et al.*, 1991). A decamer consensus sequence has been recently identified in the promoters of the *MAG*, *MGT1* and several other genes involved in the DNA repair and metabolism (Xiao *et al.*, 1993), suggesting a common mechanism of regulation. In mammalian cells induction of alkyltransferase activity has been shown in some cases, but the changes are much more modest than the ones seen in *E. Coli*, up to 10-fold. Induction of $O^6$ATase has been reported in human cancer cells after challenge with alkylating agents, but this finding has not always been confirmed (Pegg, 1990; Laval, 1990, 1991). The mechanisms underlying these increases have not been studied in detail, but they are probably at the level of transcription (Laval, 1991).

Our finding that the contents of these two DNA repair proteins, which are involved in the repair of alkylated DNA, are correlated suggests a common mechanism of regulation, and studies are currently underway in our laboratory to clarify this.

We found no correlation between the level of 3-meAde gly and the sensitivity to different alkylating agents, methylating agents or other alkylators with different structure and different interactions with DNA. In the case of tallimustine and CC-1065, two minor groove binders known to alkylate N3 adenine in a sequence-specific manner (Broggini *et al.*, 1994), the explanation could be that the kind of adduct formed on N3 adenine is bulky and might not be recognised and cleaved by the enzyme. For the cross-linking agents L-PAM and cDDP too, the cytotoxic lesions appear to be the cross-links between two adjacent guanines or guanines located on opposite strands, so the alkylation of N3 adenine might be relatively unimportant for their cytotoxicity. In fact it has been shown that the nucleotide excision repair plays an important role in the repair of the lesions caused by cDDP and L-PAM treatment (Lee *et al.*, 1993; Larminat and Bohr, 1994).

For methylating agents (DMS and MNNG) the lack of correlation between the levels of the DNA repair protein and the sensitivity confirmed previous findings (Imperatori *et al.*, 1994) on murine cells expressing the *E. Coli tag* or human *MPG* gene, which did not become resistant to the toxic effects of methylating agents. As already proposed, the endogenous levels of 3-meAde gly might be the crucial factor governing the sensitivity to methylating treatment. In the ten lines selected for this study, the 3-meAde gly contents were relatively high, possibly sufficient *per se* to repair the 3-meAde adduct caused by treatment with alkylating agents. In fact, resistance to methylating treatment has been reported in cell lines transfected with the *tag* gene, with an increase in the level of repair activity from 0.6 to 4.3 fmol $mg^{-1}$ protein $min^{-1}$ (O'Connor and Laval, 1990).

The other major factor for resistance to methylating agents is the cellular content of $O^6$ATase. However, no correlation between the transferase activity and cellular sensitivity to different alkylating agents, particularly with MNNG and DMS, which are known to cause the highest proportion of $O^6$meGua was found. It has already been shown that the sensitivity to MNNG is not necessarily associated with $O^6$ATase level (Lefebvre and Laval, 1993). Recently a defective mismatch repair has also been implicated in resistance and tolerance to some alkylating agents (such as MNU and MNNG). In this case $O^6$-meGua persists in tolerant cells but is no longer lethal. The current underlying hypothesis is that methylation tolerance arises through the loss of a mismatch repair system that is defective in many tolerant cells (Karran and Bignami, 1994).

In conclusion, the present findings suggest that the two repair enzymes, 3meAde gly and $O^6$ATase, are not necessarily vital to the sensitivity to various alkylating agents. It is likely that after DNA damage a cascade of events occurs, resulting in a certain degree of survival. For example, two cell lines may have the same level of DNA damage and the same rate of adduct removal but the activation of different pathways for cell survival, for example activation of different proteins involved in cell growth and survival (e.g. p53), so that no single factor is likely to be responsible for the sensitivity or resistance to a certain drug.

**Acknowledgements**

This work was supported by the CNR (National Cancer Council, Rome, Italy) Progetto Finalizzato ACRO Nos 94.01313-PF39 and 94.01318-PF39 and by the Italian Association for Cancer Research. LI is a recipient of a FIRC fellowship.

### References

BERANEK DT. (1990). Distribution of methyl and ethyl adducts following alkylation with monofunctional alkylating agents. *Mutat. Res.*, **231**, 11–30.

BJELLAND S AND SEEBERG E. (1987). Purification and characterization of 3-methyladenine DNA glycosylase I from *Escherichia coli*. *Nucleic Acids Res.*, **15**, 2787–2801.

BJELLAND S, BJORAS M AND SEEBERG E. (1993). Excision of 3-methylguanine from alkylated DNA by 3-methyladenine DNA glycosylase I of *Escherichia coli*. *Nucleic Acids Res.*, **21**, 2045–2049.

BOITEUX S, HUISMAN O AND LAVAL J. (1984). 3-Methyladenine residues in DNA induced the SOS function sfiA in *Escherichia coli*. *EMBO J.*, **3**, 2569–2573.

BROGGINI M, COLEY H, MONGELLI N, GRANDI M, WYATT MD, HARTLEY JA AND D'INCALCI M. (1995). DNA sequence specific adenine alkylation by the novel antitumour drug tallimustine (FCE 24517), a benzoyl nitrogen mustard derivative of distamycin. *Nucleic Acids Res.*, **23**, 81–87.

CATAPANO CV, BROGGINI M, ERBA E, PONTI M, MARIANI L, CITTI L AND D'INCALCI M. (1987). In vitro and in vivo methazolastone-induced DNA damage and repair in L-1210 leukaemia sensitive and resistant to chloroethylnitrosoureas. *Cancer Res.*, **47**, 4884–4889.

CHAKRAVARTI D, IBEANU GC, TANO K AND MITRA S. (1991). Cloning and expression in *Escherichia coli* of a human cDNA encoding the DNA repair protein *N*-methylpurine DNA glycosylase. *J. Biol. Chem.*, **266**, 15710-15715.

CHEN J AND SAMSON L. (1991). Induction of *S.cerevisiae* MAG 3-methyladenine DNA glycosylase transcript levels in response to DNA damage. *Nuclein Acids Res.*, **19**, 6427-6432.

CHEN J, DERFLER B AND SAMSON L. (1990). *Saccharomyces cerevisiae* 3-methyladenine DNA glycosylase has homology to the AlkA glycosylase of *E.coli* and is induced in response to DNA alkylation damage. *EMBO J.*, **9**, 4569-4575.

CHIRGWIN JM, PRZYBYLA AE, MACDONALD RJ AND RUTTER WJ. (1979). Isolation of biologically active ribonucleic acid from sources enriched in ribonuclease. *Biochemistry*, **18**, 5294-5299.

CITRON M, SCHOENHAUS M, GRAVER M, HOFFMAN M, LEWIS M, WASSERMAN P, NIEDERLAND M, KAHN L, WHITE A AND YAROSH D. (1993). $O^6$-methylguanine-DNA methyltransferase in human normal and malignant lung tissues. *Cancer Invest.*, **11**, 258-263.

ENGELWARD BP, BOOSALIS MS, CHEN BJ, DENG Z, SICILIANO MJ AND SAMSON LD. (1993). Cloning and characterisation of a mouse 3-methyladenine/7-methyl-guanine/3-methylguanine DNA glycosylase cDNA whose gene maps to chromosome 11. *Carcinogenesis*, **14**, 175-181.

EVENSEN G AND SEEBERG E. (1982). Adaptation to alkylation resistance involves the induction of a DNA glycosylase. *Nature*, **296**, 773-775.

FORNACE AJJ, PAPATHANASIOU MA, HOLLANDER MC AND YAROSH DB. (1990). Expression of the $O^6$-methylguanine-DNA methyltransferase gene MGMT in the $MER^+$ and $MER^-$ human tumour cells. *Cancer Res.*, **50**, 7908-7911.

GERSON SL, TREY JE, MILLER K AND BERGER NA. (1986). Comparison of $O^6$-alkylguanine-DNA alkyltransferase activity based on cellular DNA content in human, rat and mouse tissues. *Carcinogenesis*, **7**, 745-749.

HABRAKEN Y AND LAVAL F. (1993). Increased resistance of the Chinese hamster mutant *irs1* cells to monofunctional alkylating agents by transfection of the *E. coli* or mammalian N3-methyladenine-DNA- glycosylase genes. *Mutat. Res.*, **293**, 187-195.

IBEANU G, HARTENSTEIN B, DUNN WC, CHANG LY, HOFFMAN E, COQUERELLE T, MITRA S AND KAINA B. (1992). Overexpression of human DNA repair protein *N*-methylpurine-DNA glycosylase results in the increased removal of *N*-methylpurines in DNA without a concomitant increase in resistance to alkylating agents in Chinese hamster ovary cells. *Carcinogenesis*, **13**, 1989-1995.

IMPERATORI L, DAMIA G, TAVERNA P, GARATTINI E, CITTI L, BOLDRINI L AND D'INCALCI M. (1994). 3T3 NIH murine fibroblasts and B78 murine melanoma cells expressing the *Escherichia coli* N3-methyladenine-DNA glycosylase I do not become resistant to alkylating agents. *Carcinogenesis*, **15**, 533-537.

KARRAN P AND BIGNAMI M. (1994). DNA damage tolerance, mismatch repair and genome instability. *Bioessay*, **16**, 833-839.

KARRAN P, HJELMGREN T AND LINDAHL T. (1982). Induction of a DNA glycosylase for *N*-methylated purines is part of the adaptive response to alkylating agents. *Nature*, **296**, 770-773.

KAT A, THILLY WG, FANG WH, LONGLEY MJ, LI GM AND MODRICH P. (1993). An alkylation-tolerant, mutator human cell line is deficient in strand-specific mismatch repair. *Proc. Natl Acad. Sci. U.S.A.*, **90**, 6424-6428.

KLUNGLAND A, FAIRBAIRN L, WATSON AJ, MARGISON GP AND SEEBERG E. (1992). Expression of the *E. coli* 3-methyladenine DNA glycosylase I gene in mammalian cells reduced the toxic and mutagenic effects of methylating agents. *EMBO J.*, **11**, 4439-4444.

LARMINAT F AND BOHR VA. (1994). Role of the human ERCC-1 gene in gene-specific repair of cisplatin-induced DNA damage. *Nucleic Acids Res.*, **22**, 3005-3010.

LAVAL F. (1990). Induction of proteins involved in the repair of alkylated bases in mammalian cells by DNA-damaging agents. *Mutat. Res.*, **233**, 211-218.

LAVAL F. (1991). Increase of $O^6$-methylguanine-DNA-methyltransferase and N3-methyladenine glycosylase RNA transcripts in rat hepatoma cells treated with DNA-damaging agents. *Biochem. Biophys. Res. Commun.*, **176**, 1086-1092.

LAVAL J. (1977). Two enzymes are required from strand incision in repair of alkylated DNA. *Nature*, **269**, 829-832.

LEE KB, PARKER RJ, BOHR V, CORNELISON T AND REED E. (1993). Cisplatin sensitivity/resistance in UV repair-deficient Chinese hamster ovary cells of complementation groups 1 and 3. *Carcinogenesis*, **14**, 2177-2180.

LEFEBVRE P AND LAVAL F. (1993). A human cell line proficient in $O^6$-methylguanine-DNA-methyltransferase and hypersensitive to alkylating agents. *Carcinogenesis*, **14**, 1671-1675.

LINDAHL T. (1976). New class of enzymes acting on damaged DNA. *Nature*, **259**, 64-66.

LINDAHL T, SEDGWICK B, SEKIGUCHI M AND NAKABEPPU Y. (1988). Regulation and expression of the adaptive response to alkylating agents. *Annu. Rev. Biochem.*, **57**, 133-157.

LOVELESS A. (1969). Possible relevance of O-6 alkylation of deoxyguanosine to the mutagenicity and carcinogenicity of nitrosamines and nitrosamides. *Nature*, **223**, 206-207.

MARGISON GP AND O'CONNOR PJ. (1990). *Handbook of Experimental Pathology 94/1*. pp. 547-571. Springer: Heidelberg.

MATIJASEVIC Z, BOOSALIS M, MACKAY W, SAMSON L AND LUDLUM DB. (1993). Protection against chloroethylnitrosourea cytotoxicity by eukaryotic 3-methyladenine DNA glycosylase. *Proc. Natl Acad Sci. U.S.A.*, **90**, 11855-11859.

MEHTA JR, LUDLUM DB, RENARD A AND VERLY WG. (1981). Repair of $O^6$-ethylguanine in DNA by a chromatin fraction from rat liver: transfer of the ethyl group to an acceptor protein. *Proc. Natl Acad. Sci. U.S.A.*, **78**, 6766-6770.

NAKABEPPU Y, KONDO H AND SEKIGUCHI M. (1984). Cloning and characterization of the *alkA* gene of *Escherichia coli* that encodes 3-methyladenine DNA glycosylase II. *J. Biol. Chem.*, **259**, 13723-13729.

O'CONNOR TR AND LAVAL F. (1990). Isolation and structure of a cDNA expressing a mammalian 3-methyladenine-DNA glycosylase. *EMBO J.*, **9**, 3337-3342.

OLSSON M AND LINDAHL T. (1980). Repair of alkylated DNA in *Escherichia coli*. Methyl group transfer from $O^6$-methylguanine to a protein cysteine residue. *J. Biol. Chem.*, **255**, 10569-10571.

PEGG AE. (1990). Mammalian $O^6$-alkylguanine-DNA alkyltransferase: regulation and importance in response to alkylating carcinogenic and therapeutic agents. *Cancer Res.*, **50**, 6119-6129.

PEGG AE AND BYERS TL. (1992). Repair of DNA containing $O^6$-alkylguanine. *FASEB J.*, **6**, 2302-2310.

PENDLEBURY A, FRAYLING IM, SANTIBANEZ KOREF MF, MARGISON GP AND RAFFERTY JA. (1994). Evidence of the simultaneous expression of alternatively spliced N-glycosylase transcripts in human tissues and cells. *Carcinogenesis*, **15**, 2957-2960.

SAKUMI K, NAKABEPPU Y, YAMAMOTO Y, KAWABATA S, IWANAGA S AND SEKIGUCHI M. (1986). Purification and structure of 3-methyladenine-DNA glycosylase I of *Escherichia coli*. *J. Biol. Chem.*, **261**, 15761-15766.

SAMSON L, DERFLER B, BOOSALIS M AND CALL K. (1991). Cloning and characterization of a 3-methyladenine DNA glycosylase cDNA from human cells whose gene maps to chromosome 16. *Proc. Natl Acad. Sci. U.S.A.*, **88**, 9127-9131.

WALKER MC, MASTERS JR AND MARGISON GP. (1992). $O^6$-alkylguanine-DNA-alkyltransferase activity and nitrosourea sensitivity in human cancer cell lines. *Br. J. Cancer*, **66**, 840-843.

WIELSTER O, KLEIHUES P AND PEGG AE. (1984). $O^6$-alkylguanine-DNA alkyltransferase activity in human brain and brain tumours. *Carcinogenesis*, **5**, 121-124.

XIAO W AND SAMSON L. (1992). The *Saccharomyces cerevisiae* MGT1 DNA repair methyltransferase gene: its promoter and entire coding sequence, regulation and *in vivo* biological functions. *Nucleic Acids Res.*, **20**, 3599-3606.

XIAO W, DERFLER B, CHEN J AND SAMSON L. (1991). Primary sequence and biological functions of a *Saccharomyces cerevisiae* $O^6$-methylguanine/$O^4$-methylthymine DNA repair methyltransferase gene. *EMBO J.*, **10**, 2179-2186.

XIAO W, SINGH KK, CHEN B AND SAMSON L. (1993). A common element involved in transcriptional regulation of two DNA alkylation repair genes. (MAG and MGT1) of *Saccharomyces cerevisiae*. *Mol. Cell Biol.*, **13**, 7213-7221.